# Perceived COVID-19 stress and online aggression among Chinese first-year college students: a moderated mediation model

**DOI:** 10.3389/fpsyt.2023.1221379

**Published:** 2023-07-21

**Authors:** Lingjing Guo, Liyuan Xu, Qiang Yang

**Affiliations:** ^1^Center of Mental Health Education and Research, Preschool Education Research Center, School of Psychology, School of Education, Jiangxi Normal University, Nanchang, China; ^2^Mental Health Education Center, Chengdu University, Chengdu, China; ^3^Mental Health Education and Counselling Center, Department of Student Affairs Management, Beijing Sport University, Beijing, China

**Keywords:** perceived COVID-19 stress, anxiety, perceived anonymity, online aggression, first-year college students

## Abstract

**Purpose:**

Few studies have explored factors that may account for potential mechanisms between perceived coronavirus disease 2019 (COVID-19) stress and online aggression. The current study examined a moderated mediation model with anxiety as a mediator and perceived anonymity as a moderator.

**Methods:**

A cross-sectional study was conducted. 3,069 participants across China completed scales assessing perceived COVID-19 stress, anxiety, online aggression, and perceived anonymity.

**Results:**

Perceived COVID-19 stress was positively related to online aggression. The association between perceived COVID-19 stress and online aggression was mediated by anxiety. Besides, the relationship between perceived COVID-19 stress and online aggression, as well as the relationship between anxiety and online aggression were moderated by perceived anonymity.

**Conclusion:**

This study explains the possible potential mechanisms for reducing online aggression in the context of COVID-19. In order to intervene in online aggression, psychological strategies are supposed to be drawn to reduce anxiety and perceived anonymity.

## 1. Introduction

From 13 March to 9 April 2023, 3 million new cases and over 23,000 deaths were reported globally, indicating a declining overall trend (1). However, the report from World Health Organization (WHO) revealed that there had been a significant increase in some regions (1). The coronavirus disease 2019 (COVID-19) epidemic has an ongoing psychological impact on individuals (2–4). COVID-19-related policies (e.g., social distancing and lockdown) have facilitated a shift from offline to online personal life and work. The growth of the internet has enabled people to use online tools to cope with work and academic difficulties, but it has also exacerbated another serious problem: online aggression (5). Prior research revealed that the overall prevalence of online aggression among Chinese college students is 59.47% (6), and COVID-19-related restrictions and influences were related to stronger cyberbullying perpetration (7, 8). First-year students may be severely affected by the epidemic, with evidence of adverse effects of COVID-19 reported by first-year students, many with anxiety, worry, and daily life disruptions (9). Besides, first-year students are in the transition period from high school to university and are at high risk of maladjustment and poor ability to cope with stress (e.g., perceived COVID-19 stress and academic stress, 9).

People who experience cyberbullying may have more internalizing problems (e.g., anxiety and depression), while cyberbullies may be associated with more externalizing problems (e.g., disciplinary violations) (10, 11). Nevertheless, a meta-analysis suggests that internalizing problems (e.g., anxiety) are significant predictors of cyberbullying perpetration (12). Aggression is generally manifested in two forms: instrumental aggression (e.g., individuals are not threatened or hurt and initiate aggressive behavior to gain benefits) and reaction aggression (e.g., individuals are threatened or hurt and engage in retaliatory aggressive behavior) (13, 14). The study focuses on instrumental aggression as it drives people to use aggression to reach goals and achieve benefits, with greater social harm and moral impact. In addition, Zimbardo’s deindividuation theory (15) and Barlett and Gentile’s (16) learning-based model in cyberbullying perpetration illustrate that individuals’ unethical behavior (e.g., online aggression) is associated with low anonymity, and the two may reinforce each other.

We draw the General Aggression Model (GAM, 17) and deindividuation theory (15) to understand the relationship among perceived COVID-19 stress, anxiety, perceived anonymity, and online aggression among Chinese freshmen college students. To our knowledge, few studies exist on the relationship among the above variables. The present study aims to explain the potential mechanisms of perceived COVID-19 stress on online aggression through anxiety and discusses the moderating role of anonymity.

### 1.1. Perceived COVID-19 stress and online aggression

Although there is no uniform definition of online aggression (Some scholars also refer to it as “cyberbullying”), at its core, it is the act of using electronic technologies against individuals or groups of individuals on the Internet and mobile phone networks to cause harm, which the target seeks to avoid (14, 17). Anderson and Bushman’s general model of aggression (18) suggests that personal and situational factors (both referred to as input variables) influence the occurrence of aggression through present internal states (including cognition, affect, and arousal), and Kowalski et al. (17) further explain cyberbullying encountering through this theoretical model. According to the general aggression model, perceived stress is an important personal factor affecting individuals’ cognitive and affective states (19, 20). Previous research has shown a strong association between perceived stress and aggression involving adolescents (21), and youth are more likely to engage in bullying behavior (both traditional and online) to respond to stressful life events (22). Empirical evidence supports that stress is significantly associated with verbal aggression and anger (which have a closer relationship with online aggression) (23).

The COVID-19 epidemic is a stressful life event that may be associated with greater aggression in individuals. In the study, perceived COVID-19 stress is defined as the extent to which individuals perceive their lives to be unpredictable, uncontrollable, and overloaded during the COVID-19 epidemic (24, 25). Research has shown that increased perceived stress during the epidemic stimulates aggressive tendencies (26) and is associated with more cyberbullying perpetration (27). Therefore, we proposed that individuals who perceived COVID-19 stress would have more online aggression.

### 1.2. Anxiety as a mediator

A longitudinal study shows that the percentage of individuals with clinically elevated generalized anxiety was 20% before the outbreak, but rose to 40.4% after the outbreak (28). Previous research finds that COVID-19-related stress has increased the likelihood of mental health issues like anxiety (2). One possible explanation is that perceived COVID-19 stress is a control loss over one’s life and may induce anxiety in individuals. Although few studies have directly addressed the relationship between anxiety and online aggression, researchers suggest that anxiety is a precursor to aggression (29), and has a significant and positive correlation with both traditional and online aggression (30, 31). Gu et al. (30) suggest that anxiety may stimulate individuals’ sensitivity to negative emotions and amplify their negative experiences, thereby showing an increased frequency of aggression.

Under the general aggression model, COVID-19 stress is considered an input variable that may further lead to the development of individuals’ online aggression by influencing their internal states like anxiety (17, 18). In other words, individuals who perceived more COVID-19 stress could experience more anxiety, which may increase their online aggression. Those with higher levels of anxiety are more attentive to negative information and more likely to have impulsive actions (18). Therefore, we proposed that anxiety mediates the relationship between perceived COVID-19 stress and online aggression.

### 1.3. Perceived anonymity as a moderator

Perceived anonymity is defined as the degree to which individuals perceive themselves and others as anonymous in cyberspace (32). The deindividuation theory suggests that deindividuation refers to the loss of individuation felt by individuals in groups, where their self-control is diminished or absent, which may lead to unconventional antisocial behavior (15, 33). Perceived anonymity is highly correlated with individuals’ level of deindividuation, and high anonymity in the online world makes individuals unrestrained, less responsible, and more likely to engage in online aggression (33). The Barlett Gentile Cyberbullying Model (BGCM, 16) and related research (34) support that perceived anonymity is positively related to antisocial online behavior (e.g., online aggression). Furthermore, in the online context, individuals are influenced to develop aggressive urges when the ‘instigation’ factors associated with aggressive risk are activated, and perceived anonymity acts as an ‘impellance’ factor for aggressive urges, facilitating this influence and increasing the likelihood of individuals cyberbullying others (35, 36). This means that individuals with high perceived anonymity are more likely to exhibit more online aggression if influenced by input variables associated with aggressive tendencies, such as experiencing stressful life events and anxiety. Specifically, compared to individuals with low perceived anonymity, the effects of perceived COVID-19 stress on online aggression are stronger among individuals with high perceived anonymity. Similarly, the relationship between anxiety and online aggression was stronger in individuals with high perceived anonymity than in individuals with low perceived anonymity. Thus, we proposed that perceived anonymity moderated the relationship between perceived COVID-19 stress and online aggression and the relation between anxiety and online aggression.

### 1.4. The present study

**Figure 1:**
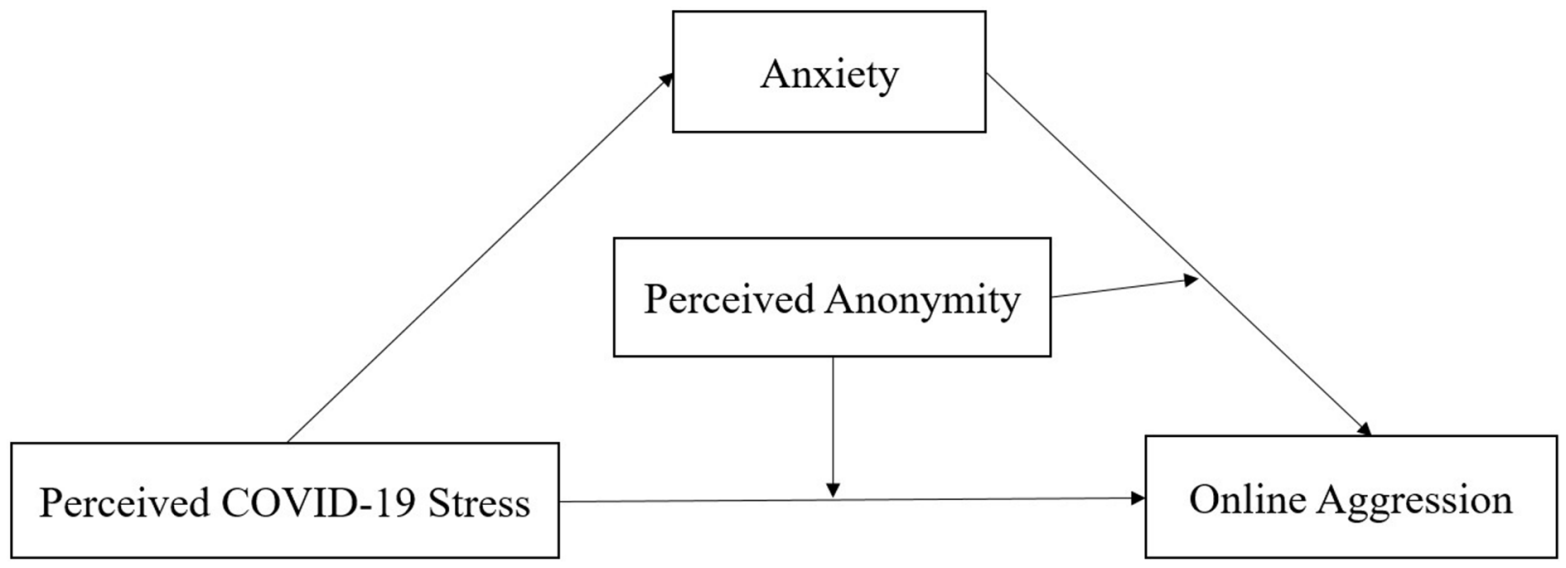
The conceptual moderated mediation model.

Previous research has provided evidence of the significant correlations between stress and online aggression. However, no study investigated the relationship between perceived COVID-19 stress and online aggression among Chinese first-year college students. We also discussed the underlying correlation mechanism between perceived COVID-19 stress and online aggression. Based on the literature review, we proposed a conceptual moderated mediation model (see Figure 1) and the following hypotheses:

*Hypothesis 1*: Perceived COVID-19 stress was positively associated with online aggression.

*Hypothesis 2*: Anxiety mediated the relationship between perceived COVID-19 stress and online aggression.

*Hypothesis 3*: Perceived anonymity would moderate the relationship between perceived COVID-19 stress and online aggression.

*Hypothesis 4*: Perceived anonymity would moderate the relationship between anxiety and online aggression.

## 2. Materials and methods

### 2.1. Participants

3,069 participants (*M*_age_ = 18.53, *SD*_age_ = 0.70) were all first-year college students and were recruited from around China. The data was collected from December 2 to December 11, 2022, when China was still in the midst of the COVID-19 epidemic. 42.31% of the participants were male, and 67.61% of the respondents reported that they were from rural areas, while 32.39% were from urban areas.

### 2.2. Measures

#### 2.2.1. Perceived COVID-19 stress

Perceived COVID-19 stress was measured by the Coronavirus Stress Measure [CSM, (37)], a well-proven questionnaire with good reliability and validity among Chinese college students (25). The unidimensional questionnaire contains 5 questions on a five-point Likert scale ranging from 0 (never) and 4 (very often) (e.g., “Due to coronavirus, how often have you felt that you were unable to control the important things in your life?”). The higher the score, the higher the perceived COVID-19 stress. In the present study, Cronbach’s alpha coefficient of this scale was good (α = 0.95).

#### 2.2.2. Online aggression

Online aggression was measured by the instrumental aggression subscale (which was focused on proactive online aggression) of the Chinese version of the Adolescent Online Aggressive Behavior Scale (AOABS, 14). The subscale consists of 15 items and college students rated each item (e.g., “I deliberately disclose other’s private information on the internet”) on a four-point scale ranging from 1(never) to 4(always). Higher scores represent a higher level of online aggression. For the current study, Cronbach’s alpha coefficient of this scale was good (*α* = 0.99).

#### 2.2.3. Anxiety

Anxiety was measured by the anxiety subscale from the Chinese short version of the Depression Anxiety and Stress Scale (DASS-21, 38). The Chinese version of DASS-21 has demonstrated good construct validity and high internal consistency among Chinese college students (38). The anxiety subscale consists of 7 items (e.g., “I felt scared without any good reason”). Respondents rated each item using a four-point scale, ranging from 0 (did not apply to me at all) to 3 (applied to me very much). Higher scores indicate individuals’ higher levels of anxiety. In the current study, Cronbach’s alpha coefficient of this scale was good (*α* = 0.93).

#### 2.2.4. Perceived anonymity

Perceived anonymity was measured by the Chinese version of the Perceived Anonymity Scale, originally developed by Jung et al. (32) and revised by Niu et al. (39) in Chinese college students. The unidimensional scale contains 4 items (e.g., “People cannot identify true-me from my message in Cyworld”) with a seven-point scale ranging from 1 (totally disagree) to 7 (totally agree). A higher score indicates a higher perceived anonymity of the Cyworld, and it is easier for individuals to hide their true selves in Cyworld. In the current study, Cronbach’s alpha coefficient of this scale was good (*α* = 0.89).

### 2.3. Data analysis

We standardized all the data before proceeding with the data analysis. We used SPSS 26 to inspect descriptive statistics and correlations among variables in the preliminary analyses. Then, Hayes’s (40, 41) PROCESS macro Model 4 for SPSS was used to test the mediating role of anxiety, and PROCESS macro Model 15 for SPSS was used to test the moderating role of perceived anonymity. 5,000 random sample bootstrapping confidence intervals (CIs) were conducted to test the moderated mediation model with a 95% confidence interval that does not include zero implying a significant effect.

## 3. Results

### 3.1. Description statistics and correlations analyses

The descriptive statistics and correlations of all the variables were illustrated in Table 1. All major variables were positively correlated with each other. Specifically, perceived COVID-19 stress was positively related to anxiety and online aggression. Besides, anxiety was positively correlated with online aggression, while perceived anonymity was positively related to perceived COVID-19 stress, anxiety, and perceived anonymity, respectively.

**Table 1:** Descriptive statistic and correlation coefficients.

Variables	*M*	*SD*	1	2	3	4
1. Perceived COVID-19 stress	6.07	4.98	1			
2. Anxiety	4.29	4.73	0.64^**^	1		
3. Online aggression	19.28	9.40	0.51^**^	0.69^**^	1	
4. Perceived anonymity	17.84	5.59	0.13^**^	0.18^**^	0.17^**^	1

*N* = 3,609, ^**^p < 0.001.

### 3.2. Testing for the mediation effect

The results of the linear analysis and mediation model were both illustrated in Table 2. Linear analysis of SPSS was used to test hypothesis 1 that perceived COVID-19 stress would be positively related to online aggression. The results showed that perceived COVID-19 stress was significantly positively related to online aggression (*β* = 0.41, *p*<0.001), which supported hypothesis 1. Then, we used Model 4 of the PROCESS macro to test hypothesis 2 that the effects of perceived COVID-19 stress on online aggression would be mediated by anxiety. Results showed that perceived COVID-19 stress was significantly related to anxiety (*β* = 0.61, *p*<0.001), and online aggression (*β* = 0.12, *p*<0.001). In addition, anxiety was significantly related to online aggression (*β* = 0.48, *p*<0.001). The indirect effects of perceived COVID-19 stress on online aggression through anxiety were significant (*β* = 0.33, 95% *CI* = [0.30, 0.36]), which supported hypothesis 2. Furthermore, after adding anxiety to the regression equation in Model 3 compared to Model 1(see Table 2), perceived COVID-19 stress remained significantly associated with online aggression, indicating a partial mediating effect of anxiety.

**Table 2:** Testing mediation effects of perceived COVID-19 stress on online aggression.

Predictors	Model 1 (OA)		Model 2 (AN)		Model 3 (OA)		Model 5 (OA)	
	*β* (95% CI)	*t*	*β* (95% CI)	*t*	*β* (95% CI)	*t*	*β* (95% CI)	*t*
Gender	−0.63 (−0.68, −0.58)	35.22^**^	−0.48 (−0.53, −0.43)	−19.38^**^	−0.40 (−0.44, −0.36)	−18.46^**^	−0.40 (−0.44, −0.36)	−19.22^**^
PCS	0.41 (0.39, 0.44)	−26.46^**^	0.61 (0.59, 0.64)	50.16^**^	0.12 (0.10, 0.15)	9.12^**^	0.10 (0.07, 0.12)	7.56^**^
AN					0.48 (0.45, 0.50)	34.36^**^	0.39 (0.37, 0.42)	28.27^**^
PA							0.07 (0.05, 0.09)	6.89^**^
PCS × PA							0.05 (0.02,0.07)	3.84^**^
AN × PA							0.13 (0.11, 0.17)	10.47^**^
*R^2^*	0.47		0.38		0.53		0.58	
*F*	1576.11^**^		1090.05^**^		1357.96^**^		813.66^**^	

*N* = 3,609, ^**^p < 0.001; PCS, Perceived COVID-19 stress; AN, Anxiety; OA, Online Aggression; PA, Perceived Anonymity.

### 3.3. Perceived anonymity as a moderator

The results of the moderation effects of perceived anonymity were illustrated in Table 2. We adopted the Model 15 of the PROCESS macro to test the moderation effect of perceived anonymity between perceived COVID-19 stress and online aggression. Results illustrated that the interaction between perceived COVID-19 stress and perceived anonymity was significantly related to online aggression (*β* = 0.05, *p* < 0.001, 95% *CI* = [0.02, 0.07]). The interaction between anxiety and perceived anonymity was significantly related to online aggression (*β* = 0.13, *p* < 0.001, 95% *CI* = [0.11, 0.17]). Thus, perceived anonymity moderated the direct and indirect pathways of perceived COVID-19 stress on online aggression.

**Figure 2:**
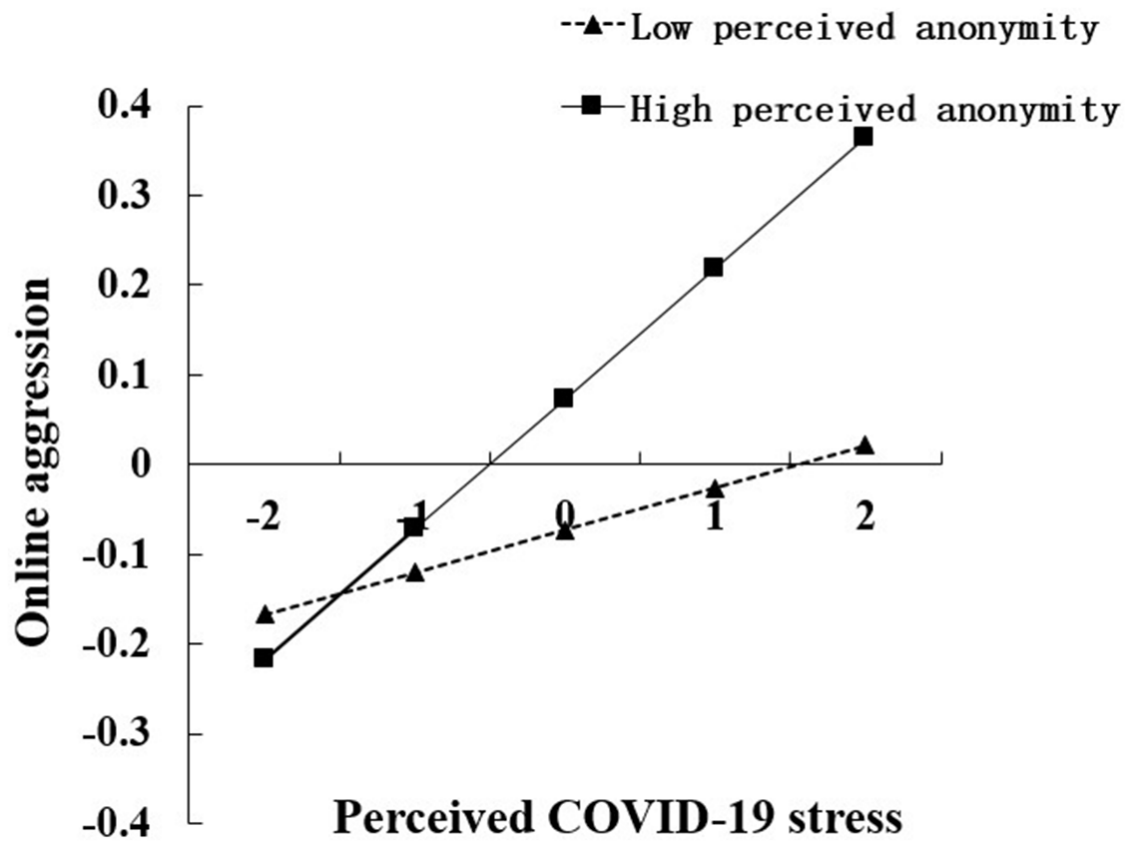
Interaction effect of perceived COVID-19 stress and perceived anonymity on online aggression.

**Figure 3:**
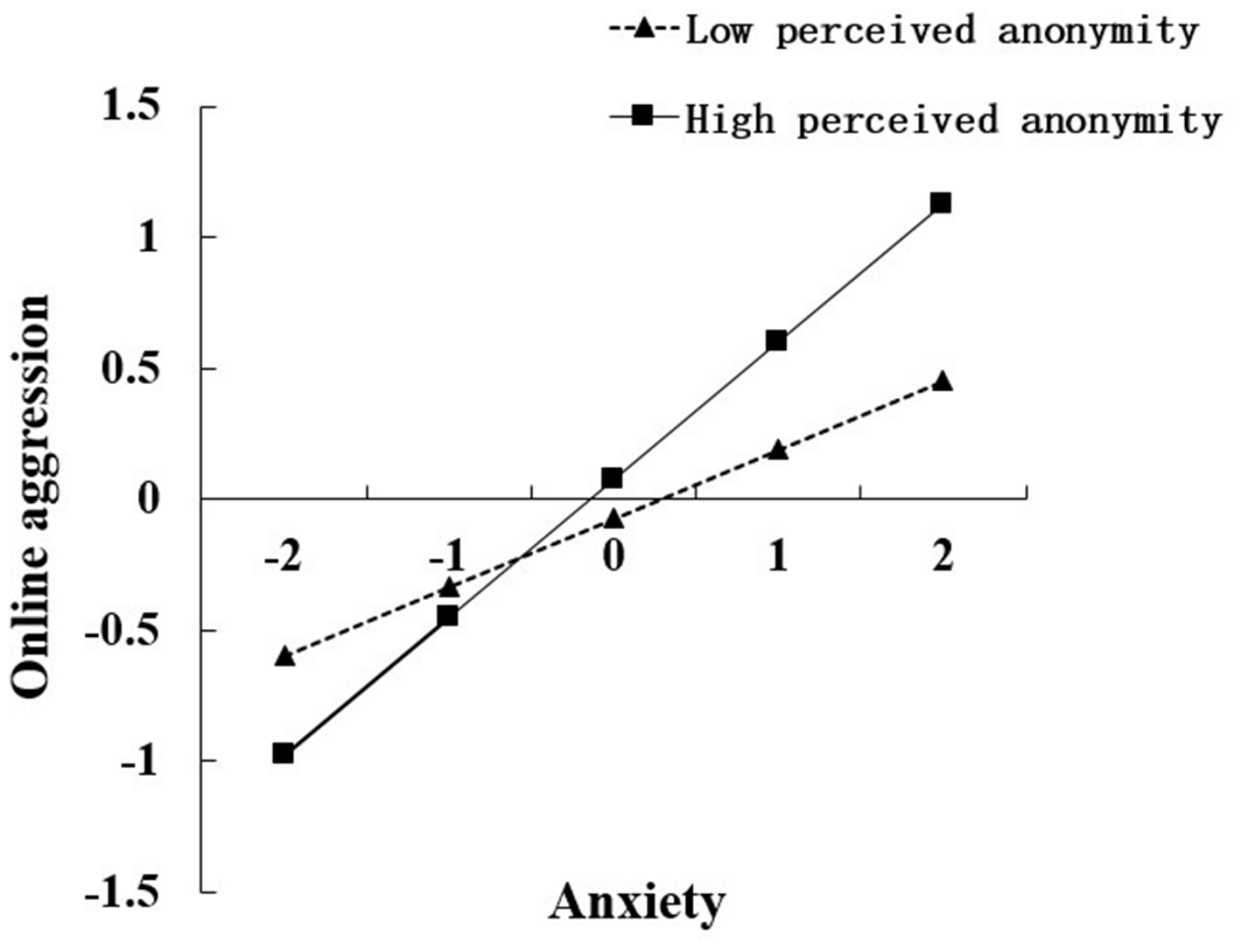
Interaction effect of anxiety and perceived anonymity on online aggression.

We conducted simple slope tests to visualize the interaction patterns. We plotted figures of perceived COVID-19 stress against online aggression (see Figure 2) and anxiety against online aggression (see Figure 3) under high and low (±1 SD from the mean) levels of perceived anonymity, respectively. The results of simple slope tests suggested that perceived COVID-19 stress was significantly related to online aggression for college students with high perceived anonymity (*β*_high PA_ = 0.14, *p*<0.001, 95% *CI* = [0.11, 0.18], see Figure 2) and those with low perceived anonymity (*β*_low PA_ = 0.05, *p* = 0.005<0.01, 95% *CI* = [0.02, 0.07], see Figure 2). However, compared to low perceived anonymity students, the effects of perceived COVID-19 stress on online aggression were stronger among high perceived anonymity students. In addition, the results of simple slope tests also suggested that anxiety was significantly positively related to online aggression for both college students with low and high perceived anonymity (*β*_high PA_ = 0.52, *p*<0.001, 95% *CI* = [0.49, 0.55]; *β*_low PA_ = 0.27, *p*<0.001, 95% *CI* = [0.23, 0.31]; see Figure 3). In other words, compared to college students with low perceived anonymity, those with high perceived anonymity would be more likely to be influenced by anxiety and to have more online aggression.

## 4. Discussion

The present study aims to discuss the effects of perceived COVID-19 stress on online aggression. The results found that perceived COVID-19 stress was positively significantly related to Chinese freshman college students’ online aggression. This study further constructed a moderated mediation model to probe the mechanism of perceived COVID-19 stress on online aggression. The results showed that anxiety mediated the association between perceived COVID-19 stress and online aggression, and perceived anonymity was a moderator between the perceived COVID-19 stress and online aggression and between anxiety and online aggression.

### 4.1. Perceived COVID-19 stress and online aggression

No previous studies have discussed the relationship between perceived COVID-19 stress and online aggression among first-year college students; this study found that more perceived COVID-19 stress among first-year students may be associated with more online aggression, supporting hypothesis 1, which is consistent with the adult population (27) and international student populations (2). Results from neurological studies also support that perceived stress is significantly associated with individuals’ aggressive behavior (20). The psychological changes involved in the shift to university are an important life transition, accompanied by changes in several important areas of life, including school, social life, and family life, where new students need to adapt to their new environment, establish new relationships, and learn to take personal responsibility (42).

During the COVID-19 epidemic, freshmen face not only the usual life changes but also interpersonal problems (the lock-down policies reduce peer interpersonal communication) and learning problems (difficulties with online learning and the transition from centralized to independent and intense learning) caused by the unstable epidemic (43). Thus, freshmen face multiple stressors due to life transitions and stressors related to the epidemic.

The more stressors first-year students are exposed to, the more likely they are to use the Internet to solve or escape stress-related problems (23, 44), which also increases their risk of online aggression due to their enhanced access to the Internet (5). The results of this study support Kowalski et al.’s (17) views on the use of the general aggression model to understand online aggression and validate that perceived COVID-19 stress is an important individual input variable influencing freshman online aggression.

### 4.2. The mediating role of anxiety

After examining the relationship between perceived COVID-19 stress and online aggression, this study further examined the mediating role of anxiety between the two variables. This study found that anxiety was an important mediator between perceived COVID-19 stress and individuals’ online aggression, supporting research hypothesis 2. The results showed a significant positive relationship between perceived COVID-19 stress and anxiety, which is consistent with previous studies during epidemics (9, 43). This study collected data during the recent new outbreak in China when some universities were again shifting from offline to online teaching requiring social distance. As the unblocked status has been maintained for some time, first-year students are more likely to feel control loss and overloading on their lives (which is defined as perceived COVID-19 stress) and worries about the future when restrictions related to COVID-19 are enacted again, exacerbating the potential for mental health problems such as anxiety disorders (2). The many unconventional stressors linked to the epidemic may have primarily contributed to the large increase in anxiety disorders following the epidemic compared to the pre-epidemic period (28, 44).

In line with prior research (17, 45), the present study found a significant positive relationship between anxiety and online aggression. This could be explained by the potential increase in online aggression as anxiety may increase an individual’s propensity to process negative information and have negative processing bias when interpreting ambiguous scenes and information, which often exist in cyberspace due to the absence of context like expressions, sounds (45, 46).

We examined the postulates of Kowalski et al.’s views on the use of the general aggression model to understand online aggression (17, 18) by exploring whether anxiety is an indirect cause of the effect between perceived COVID-19 stress and online aggression, and the results supported the model. Kowalski et al.’s views illustrate that input variables, including personal and situational factors, can influence individuals’ online aggression through three direct pathways: cognitive, affective, and arousal (internal state) (17, 18). After considering the inputs and the internal state, individuals engage in an appraisal and decision-making process, ultimately choosing to act thoughtfully or impulsively. In contrast, anxious individuals tend to make impulsive decisions (47). This connection has been discussed in neurological research, which suggests that perceived stress and anxiety are risk factors for aggression, and they share to some extent the same cortical and subcortical anatomical underpinnings as aggression and that brain structures involved in anxiety symptoms also play a partially mediating role between these factors and aggression (20).

### 4.3. The moderating role of perceived anonymity

The present study further examined the moderating role of perceived anonymity in a mediated model of perceived COVID-19 stress, anxiety, and online aggression. The findings found a significant positive correlation between perceived anonymity and online aggression, consistent with previous research (36).

The results of the moderated effects analysis showed that perceived anonymity moderated the direct effect of perceived COVID-19 stress on online aggression; in particular, when the level of perceived COVID-19 stress increased, online aggression increased at a slower rate for students with low perceived anonymity, while online aggression of students with high perceived anonymity would increase at a faster rate with increasing perceived COVID-19 pressure. This implied that individuals with high perceived anonymity were more sensitive to growth in perceived COVID-19 stress compared to individuals with low perceived anonymity. Indeed, perceived anonymity moderated the effect of anxiety on online aggression. However, online aggression in both low and high perceived anonymity individuals rose with anxiety specifically, the rate of increment in the low perceived anonymity group was lower than in the high perceived anonymity group. Both of these results support the theory of deindividuation (15) and confirm the contribution of anonymity. High levels of anonymity in online social media contexts are associated with higher levels of deindividuation. Individuals are more likely to engage in online aggression incidents when influenced by input factors and the “affect” of the internal state relevant to online aggression. Alternatively, online aggression perpetrators are less likely to fear revealing their actions, as with traditional aggression, due to screen barriers (48, 49).

## 5. Limitations and directions for future research

The present study still has the following limitations. Firstly, the cross-sectional study could not account for the causal relations between variables. Future studies could investigate causal inference using longitudinal or experimental design. Secondly, this study only tested a moderated mediation model with Chinese college students, and future studies could extend the findings to groups in other cultural contexts and make cross-cultural comparisons. Thirdly, the data collected in this study were during the epidemic, which may not be applicable to samples collected during non-epidemic periods. Future studies may consider validation during non-epidemic periods. Fourthly, in validating Kowalski et al.’s (17) view on the use of the general aggression model to understand online aggression, this study focuses on only one part of the internal state phase of the view proposed —— “affect” (e.g., anxiety). Future research may continue to test the applicability of Kowalski et al.’s views on online aggression and explore other theories that probably explain online aggression.

Despite these limitations, the present study also has theoretical and practical value. Theoretically, this study validates Kowalski et al.’s (17) views on the use of the general aggression model to understand online aggression and deindividuation theories through a mediating model of regulation and identifies a mediating role for anxiety and a moderating role for perceived anonymity, contributing to an understanding of the mechanisms underlying the relationship between perceived COVID-19 stress and cyberattack. Practically, this study shows that anxiety is a crucial variable mediating the relationship between perceived COVID-19 stress and online aggression, that immediate blocking and moderation of individual anxiety can help reduce their levels of online aggression, and that schools and communities can monitor students’ stress and anxiety states and provide timely assessment and intervention. In addition, as the internet has become an essential social venue, schools and communities can provide online interventions and guidance on communication skills based on online platforms that are conducive to reducing students’ stress and anxiety and leading to a more positive online social orientation.

## 6. Conclusion

Anxiety is an important mediator when exploring the potential mechanisms of perceived COVID-19 stress on online aggression among first-year university students in China. Future research is recommended to consider “anxiety” more comprehensively and to extend the validation study of Kowalski et al.’s (17) views on the use of the general aggression model to understand online aggression. Besides, perceived anonymity moderated the direct pathway (perceived COVID-19 stress → online aggression) and indirect pathway (anxiety → online aggression) from perceived COVID-19 stress through anxiety to online aggression, suggesting that perceived anonymity is an important risk factor associated with the increase in online aggression. Future research may consider how to intervene in perceived anonymity and focus on clarifying the need for individuals to take responsibility for their own actions in cyberspace in order to minimize the level of perceived anonymity. Moreover, enhanced measures to alleviate stress and anxiety should be considered to lower online aggression, for example, by using online resources to assess and intervene with the degree of individuals’ stress and anxiety to reduce online aggression.

## Data availability statement

The raw data supporting the conclusions of this article will be made available by the authors, without undue reservation.

## Ethics statement

The studies involving human participants were reviewed and approved by Ethics Committee of the Jiangxi Normal University. The patients/participants provided their written informed consent to participate in this study.

## Author contributions

LG, QY, and LX: conception and design of the study. QY: supervision and project administration. QY and LX: data collection. LG: data analysis. LG and LX: original manuscript. All authors contributed to the article and approved the submitted version.

## Funding

This study was supported by the National Natural Science Foundation of China (72164018), The Humanities and Social Sciences Program of the Ministry of Education (22YJA190012), Ideological and Political Project of Beijing Sport University (No. 2022SZ009) and Jiangxi Social Science Foundation Project (21JY13).

## Conflict of interest

The authors declare that the research was conducted in the absence of any commercial or financial relationships that could be construed as a potential conflict of interest.

## Publisher’s note

All claims expressed in this article are solely those of the authors and do not necessarily represent those of their affiliated organizations, or those of the publisher, the editors and the reviewers. Any product that may be evaluated in this article, or claim that may be made by its manufacturer, is not guaranteed or endorsed by the publisher.
